# AIBP Protects Müller Glial Cells Against Oxidative Stress-Induced Mitochondrial Dysfunction and Reduces Retinal Neuroinflammation

**DOI:** 10.3390/antiox13101252

**Published:** 2024-10-17

**Authors:** Seunghwan Choi, Soo-Ho Choi, Tonking Bastola, Keun-Young Kim, Sungsik Park, Robert N. Weinreb, Yury I. Miller, Won-Kyu Ju

**Affiliations:** 1 Hamilton Glaucoma Center, Shiley Eye Institute, Viterbi Family Department of Ophthalmology, University of California San Diego, La Jolla, CA 92039, USA; seunghwan.choi@prazertx.com (S.C.); tbastola@health.ucsd.edu (T.B.); sup012@ucsd.edu (S.P.); rweinreb@health.ucsd.edu (R.N.W.); 2 Department of Medicine, University of California San Diego, La Jolla, CA 92039, USA; soc002@health.ucsd.edu (S.-H.C.); yumiller@health.ucsd.edu (Y.I.M.); 3 National Center for Microscopy and Imaging Research, Department of Neurosciences, University of California San Diego, La Jolla, CA 92039, USA; kkim@health.ucsd.edu

**Keywords:** glaucoma, neuroinflammation, retinal ganglion cells, oxidative stress, AIBP, mitochondria, Müller glia

## Abstract

Glaucoma, an optic neuropathy with the loss of retinal ganglion cells (RGCs), is a leading cause of irreversible vision loss. Oxidative stress and mitochondrial dysfunction have a significant role in triggering glia-driven neuroinflammation and subsequent glaucomatous RGC degeneration in the context of glaucoma. It has previously been shown that apolipoprotein A-I binding protein (APOA1BP or AIBP) has an anti-inflammatory function. Moreover, *Apoa1bp^−/−^* mice are characterized by retinal neuroinflammation and RGC loss. In this study, we found that AIBP deficiency exacerbated the oxidative stress-induced disruption of mitochondrial dynamics and function in the retina, leading to a further decline in visual function. Mechanistically, AIBP deficiency-induced oxidative stress triggered a reduction in glycogen synthase kinase 3β and dynamin-related protein 1 phosphorylation, optic atrophy type 1 and mitofusin 1 and 2 expression, and oxidative phosphorylation, as well as the activation of mitogen-activated protein kinase (MAPK) in Müller glia dysfunction, leading to cell death and inflammatory responses. In vivo, the administration of recombinant AIBP (rAIBP) effectively protected the structural and functional integrity of retinal mitochondria under oxidative stress conditions and prevented vision loss. In vitro, incubation with rAIBP safeguarded the structural integrity and bioenergetic performance of mitochondria and concurrently suppressed MAPK activation, apoptotic cell death, and inflammatory response in Müller glia. These findings support the possibility that AIBP promotes RGC survival and restores visual function in glaucomatous mice by ameliorating glia-driven mitochondrial dysfunction and neuroinflammation.

## 1. Introduction

Glaucoma, an optic neuropathy with the loss of retinal ganglion cells (RGCs), is a leading global cause of irreversible vision loss and blindness [1]. The common critical pathological phenotypes of this optic neuropathy include progressive optic nerve axon degeneration and RGC death, ultimately with some efficacy, but they are often inadequate to stop disease progression [1,2]. Hence, investigating the underlying pathophysiological mechanisms of glaucoma is crucial. Understanding these mechanisms could supplement IOP-lowering strategies or offer independent approaches to improve treatment efficacy.

Vision impairment in common glaucomatous retinal degenerative conditions is significantly influenced by oxidative stress, a recognized risk factor in glaucoma [3,4,5,6,7]. Therapeutic strategies targeting oxidative stress and mitochondrial dysfunction are of interest for potential glaucoma treatments. Importantly, clinical studies have linked primary open-angle glaucoma to specific mitochondrial cytochrome c oxidase subunit I of the oxidative phosphorylation (OXPHOS) complex IV [8] and single-nucleotide polymorphisms of toll-like receptor 4 (TLR4) [9]; this suggests a connection between OXPHOS stress-induced mitochondrial dysfunction and TLR4-mediated neuroinflammation in glaucoma neurodegeneration. However, the precise mechanisms linking oxidative stress, mitochondrial dysfunction, and neuroinflammation in glaucoma remain poorly understood.

Apolipoprotein A-I binding protein (AIBP; gene name APOA1BP alias also known as NAXE) is a secreted protein that regulates cholesterol removal from the plasma membrane [10,11,12]. Extracellular AIBP binds to TLR4, thereby directing cholesterol depletion to inflammatory cells with high levels of TLR4 expression [13,14]. Intracellular AIBP is localized in mitochondria and modulates mitophagy by regulating Parkin and mitofusin (MFN) 1 and MFN2. Under oxidative stress conditions, this regulatory mechanism helps eliminate damaged mitochondria in macrophages [13,14,15]. We have demonstrated that AIBP expression is significantly downregulated in mouse and human glaucomatous retina [14,15] and that AIBP deficiency induces mitochondrial dysfunction in Müller glial cells [14,15,16]. Since Müller glial cells regulate retinal neuroinflammation [14,15,16], we hypothesized that AIBP deficiency is associated with RGC damage and visual dysfunction under oxidative stress conditions.

In the present study, we found that AIBP deficiency exacerbated the oxidative stress-induced impairment of mitochondrial dynamics, biogenesis, and function in Müller glia, leading to impaired visual function. Conversely, the administration of recombinant AIBP (rAIBP) restored mitochondrial dynamics and function in the retina and improved visual function under oxidative stress. rAIBP administration reduced TLR4-associated lipid rafts, restored mitochondrial dynamics and function, decreased inflammasome activation and inflammatory response, and reduced Müller glial cell death. This protection may promote RGC survival and restore visual function by ameliorating glia-driven mitochondrial dysfunction and neuroinflammation in glaucoma.

## 2. Materials and Methods

### 2.1. Animals

C57BL/6J (The Jackson Laboratory, Bar Harbor, ME, USA) and AIBP knock-out (*Apoa1bp*^−/−^) mice were housed in covered cages, fed with a standard rodent diet ad libitum, and kept on a 12 h light/12 h dark cycle. C57BL/6J mice were bred in-house for experiments and used as wild-type (WT) mice. *Apoa1bp*^−/−^ mice on a C57BL/6J background were generated in our laboratory, as previously reported [15]. Animals were assigned randomly to experimental and control groups. Visual function tests were studied with 10-month-old male and female mice. Research in ophthalmic vision involving animals was conducted in accordance with the Association for Research in Vision and Ophthalmology Statement for the Use of Animals in Ophthalmic Vision Research and under protocols approved by the Institutional Animal Care and Use Committee at the University of California, San Diego (CA, USA). (IACUC S12063 for mouse).

### 2.2. Recombinant AIBP

N-terminal His-tagged AIBP was produced in a baculovirus/insect cell expression system to allow for post-translational modification and to ensure endotoxin-free preparation as previously described [17,18]. A bulk production of recombinant AIBP was ordered from Selvita Inc. (Kraków, Poland) and was stored at −80 °C.

### 2.3. Induction of Retinal Oxidative Stress

To induce oxidative stress, mice received intraperitoneal (IP) injections of paraquat (PQ) (15 mg/kg, Sigma-Aldrich, St. Louis, MO, USA) in saline solution three times for 1 week as previously described [19]. Measurements for visual function tests (*n* = 6 to 8 mice per group) such as pattern electroretinogram (pERG), pattern visual evoked potential (pVEP), and virtual optomotor response were assessed 1 week after PQ treatment.

### 2.4. Tissue Preparation

Mice were anesthetized by an IP injection of a mixture of ketamine (100 mg/kg, Ketaset; Fort Dodge Animal Health, Fort Dodge, IA, USA) and xylazine (9 mg/kg, TranquiVed; Vedeco, Inc., St. Joseph, MO, USA) before cervical dislocation. For immunohistochemistry (n = 3 mice per group), the retinas were dissected from the choroids and fixed with 4% paraformaldehyde (Sigma-Aldrich) in PBS (pH 7.4) for a duration of 2 h at 4 °C. Retinas were washed several times with PBS then dehydrated through graded levels of ethanol and embedded in polyester wax. For Western blot and PCR analysis, extracted retinas were immediately used.

### 2.5. Immunohistochemistry

The immunohistochemical staining of 7 μm wax sections of full-thickness retinas was performed. Sections from wax blocks from each group (*n* = 4 retinas/group) were used for immunohistochemical analysis. To prevent non-specific background, tissues were incubated in 1% bovine serum albumin (BSA, Sigma-Aldrich)/PBS for 1 h at room temperature before incubation with the primary antibodies for 16 h at 4 °C. After several wash steps, the tissues were incubated with the secondary antibodies for 4 h at 4 °C and subsequently washed with PBS. The sections were counterstained with the nucleic acid stain Hoechst 33342 (1 μg/mL; Invitrogen, Carlsbad, CA, USA) in PBS. Images were acquired using Keyence All-in-One Fluorescence microscopy (BZ-X810, Keyence Corp. of America, Itasca, IL, USA). Each target protein fluorescent integrated intensity in pixel per area was measured using the ImageJ software version 1.54i [National Institutes of Health (NIH), Bethesda, MD, USA]. All imaging parameters remained the same and were corrected with background subtraction. The primary and secondary antibodies used in this study are presented in Supplementary Table S1.

### 2.6. pERG and pVEP Analysis

pERG and pVEP were measured with a Celeris apparatus (Diagnosys, Lowell, MA, USA) as previously reported [14,15]. After dark adaptation overnight, the mice were anesthetized with intraperitoneal injections of a cocktail of ketamine/xylazine under red light, and a mixture of 0.5% tropicamide and 2.5% phenylephrine was applied directly to the eye to dilate pupils. Eyes were also treated with 1% proparacaine drops and 0.3% hypromellose gel to prevent corneal drying and cataracts. pERG responses were recorded using alternating, reversing, black and white vertical stimuli at 1 Hz (2 reversals per second) and 50 candela/m^2^ delivered by the pattern stimulator. Then, 200 traces were recorded per eye, and averaged waveforms were calculated in which amplitudes (µV) were measured from the P1 peak to the N2 trough. At the same time, pVEP responses were recorded. For all the recordings, ground and reference needle electrodes were placed subcutaneously in the tail and snout, and the active electrode was placed subdermally in the midline of the head at the location of the visual cortex. Each eye was separately exposed to 100 flashes of 1 Hz, 0.05 cd s/m^2^ white light through the corneal stimulators and recorded for 300 ms with a sample frequency of 2000 Hz. Then, 200 traces were recorded per eye, and averaged waveforms were calculated in which amplitudes (µV) were measured from the P1 peak to the N1 trough. For each mouse, we performed five trials and swept 100 times per trial. The low- and high-filter frequency cutoffs for pVEP were set to 1.25 Hz and 100 Hz. All data measurements were recorded while maintaining a constant body temperature between 37 °C and 38 °C using the system’s heat pads. The data were analyzed by the software Espion V6 (Diagnosys) [20,21].

### 2.7. Virtual Optomotor Response Analysis

Spatial frequency was analyzed on a virtual optomotor system (OptoMotry; Cerebral Mechanics Inc., Lethbridge, AB, Canada) as previously reported [15,19,22]. Unanesthetized mice were placed on an unrestricted platform in the center of a virtual cylinder comprising four monitors arranged in a square (arena) that project a sinusoidal grating (i.e., white versus black vertical bars) rotating at 12 deg/s. Mice were monitored by a camera mounted at the top of the arena, while a cursor placed on the forehead centered the rotation of the cylinder at the animal’s viewing position. To assess visual acuity, tracking was determined when the mouse stopped moving its body and only head-tracking movement was observed. The spatial frequency threshold, a measure of visual acuity, was determined automatically using the accompanying OptoMotry software (Version 2.1.0), which uses a step-wise paradigm based upon head-tracking movements at 100% contrast. Spatial frequency began at 0.042 cyc/deg, which gradually increased until head movement was no longer observed.

### 2.8. Cell Culture and rAIBP Administration

rMC-1 cells, immortalized rat retinal Müller glial cell line (Kerafast, Boston, MA, USA), were grown in Dulbecco’s Modified Eagle Medium (DMEM, Corning Inc, New York, NY, USA) supplemented with 5% fetal bovine serum and 1% penicillin/streptomycin solution at 37 °C in a humidified CO_2_ incubator. The cells were seeded into six-well plates at a density of 2 × 10^5^ cells/well and maintained for 24 h. Subsequently, the cells were pretreated with 0.2 μg/mL rAIBP or BSA as a control for 2 h, followed by stimulation with PQ (500 μM) for 24 h. A cell culture experiment was performed with 3 independent experiments.

### 2.9. siRNA Transfection

rMC-1 cells were transfected with scramble siRNA or AIBP siRNA purchased from Origene (Rockville, MD, USA) using the Amaxa Nucleofector (Lonza Group, Basel, Switzerland). Briefly, the cells (3 × 10^6^) were resuspended in 100 μL of nucleofector solution mix followed by the addition of 20 nM of scramble siRNA or AIBP siRNA and transfected according to the manufacturer’s instructions. The cells were seeded into six-well plates at a density of 5 × 10^5^ cells/well and maintained for 24 h.

### 2.10. Mitochondrial Membrane Potential (MMP) and Mitochondrial Reactive Oxygen Species (mtROS) Measurement

rMC-1 cells were seeded into 6-well plates at a density of 2 × 10^5^ cells/well, maintained for 24 h, and treated with the indicated materials for 24 h. MMP was determined by flow cytometry. The cells were incubated with TMRE solution (200 nM, Invitrogen) for 30 min at 37 °C. The intracellular mtROS level in rMC-1 cells was measured by flow cytometry. After treatment, the cells were incubated with mitoSOX solution (500 nM, Invitrogen) at 37 °C for 30 min.

### 2.11. Western Blot Analyses

Retina tissues and rMC-1 cells were homogenized on ice for 1 min using a modified RIPA lysis buffer [50 mM Tris-HCl, pH 8.0, 150 mM NaCl, 1% Nonidet P-40, 0.5% deoxycholic acid, 0.1% sodium dodecyl sulfate (SDS)], containing protease and phosphatase inhibitor cocktail (ThermoFisher Scientific, San Diego, CA, USA), and incubated on ice for 30 min for complete cell lysis. Harvested retinas were homogenized in RIPA buffer using a motorized tissue grinder (ThermoFisher Scientific). Cell and tissue debris was removed by centrifugation at 12,000× *g* for 15 min. Lysates (10 μg of protein) were separated by 4–20% Mini-PROTEAN TGX-precast protein gel electrophoresis (Bio-Rad, Hercules, CA, USA), and target protein levels were determined by Western blot analysis [19]. The membranes were blocked with 5% non-fat dry milk in PBS/0.1% Tween-20 (PBS-T) for 1 h at room temperature, then incubated with primary antibodies overnight at 4 °C. After washing several times with PBS-T, the membranes were incubated with horseradish peroxidase-conjugated goat anti-mouse or rabbit IgG (1:1000–7000; Bio-Rad; Cat# 1721011 or 1706515) for 1 h at room temperature and developed using an enhanced chemiluminescence substrate system. The images were captured and quantified using the ImageQuant™ LAS 4000 system (GE Healthcare Bio-Science, Piscataway, NJ, USA) and ImageJ software (NIH). For each experiment, we conducted multiple biological replicates (at least 3 replicates), quantified the results, calculated averages, and presented the data as bar graphs. The blots shown were carefully selected based on these quantitative data to ensure clarity and consistency.

### 2.12. Immunocytochemistry

rMC-1 cells were fixed in 4% paraformaldehyde for 15 min at room temperature. After gently washing, the cells were permeabilized with 0.1% triton X-100 and incubated with a cleaved caspase-3 antibody for 16 h at 4 °C. After three wash steps, the cells were incubated with Alexa Fluor-568 conjugated donkey anti-rabbit IgG antibody for 2 h. For nuclear staining, the cells were further incubated with Hoechst 33342 (1 μg/mL, Invitrogen) for 5 min. Images were acquired using Keyence All-in-One Fluorescence microscopy (BZ-X810, Keyence). Each target protein fluorescent integrated intensity in pixel per area was measured using the ImageJ software (NIH) as described above.

### 2.13. Quantitative Real-Time PCR (qRT-PCR) Analysis

Total mRNAs were isolated from rMC-1 using a Trizol reagent. cDNAs were prepared from 1 μg of RNA using a SuperScript III First-strand synthesis system (Invitrogen). qRT-PCR was performed with the PowerUp SYBR master mix (Applied Biosystems, Forster City, MA, USA) according to the manufacturer’s instructions. The mRNA levels of target genes were determined and quantitated using their specific primers and normalized to glyceraldehyde-3-phosphate dehydrogenase (GAPDH) [23]. The primers used in this study are presented in Supplementary Table S2.

### 2.14. Oxygen Consumption Rate (OCR) and Extracellular Acidification Rate (ECAR) Analyses

rMC-1 cells (5 × 10^4^ per well) were seeded into Seahorse XF24-well plates. At 24 h, cells were pretreated with either BSA or rAIBP (0.2 μg/mL) for 2 h, followed by exposure to PQ (50 μM). OCR and ECAR were measured using an XF24 Extracellular Flux analyzer (Agilent, La Jolla, CA, USA). For OCR analysis, we used a Seahorse XF cell mito stress test kit (Agilent) as previously reported [5,24,25]. After measuring basal respiration, oligomycin (2 μg/mL, Sigma-Aldrich), an inhibitor of ATP synthesis; carbonyl cyanide 4-(trifluoromethoxy) phenylhydrazone (FCCP; 1 μM, Sigma-Aldrich), the uncoupler; and rotenone (2 μM, Sigma-Aldrich), an inhibitor of mitochondrial complex I, were sequentially added to measure maximal respiration, ATP-linked respiration, and spare respiratory capacity. For ECAR analysis, we used a Seahorse XF cell glycolysis stress test kit (Agilent). Glucose (10 mM, Sigma-Aldrich), oligomycin (1 μM, Sigma-Aldrich), and 2-deoxyglucose (2-DG, 50 mM, Sigma-Aldrich) were sequentially added to measure glycolysis, glycolytic capacity, and glycolytic reserve.

### 2.15. Immunofluorescence Staining of TLR4-Associated Lipid Rafts

After exposure to oxidative stress, rMC-1 cells were immediately put on ice, washed once with cold PBS, and fixed with 4% PFA for 10 min. Cells were washed twice with cold PBS and incubated with blocking buffer containing 5% FBS for 30 min without permeabilization, followed by staining with Cholera Toxin B (CTxB)-Alexa Fluor 594 (Invitrogen) to stain lipid rafts and rabbit anti-TLR4 antibody (Proteintech, Rosemont, IL, USA) for 2 h at room temperature, washed and incubated with anti-rabbit Alexa Fluor 647 conjugated secondary antibody (Invitrogen) for 1 h at room temperature. Cells were washed five times, and coverslips were mounted with Prolong Gold with DAPI (Invitrogen) into slides. Image acquisition was conducted using Keyence All-in-One Fluorescence microscopy (BZ-X810, Keyence), and image analysis was performed using ImageJ software (NIH). A colocalization assessment was executed using the colocalization finder plugin, facilitating the calculation of Pearson’s coefficients.

### 2.16. Statistical Analysis

For comparison between two groups that have a small number of samples related to a fixed control, statistical analysis was conducted utilizing nonparametric analysis and a one-sample *t*-test. For comparison between two independent groups, a two-tailed Student’s *t*-test was performed. For multiple group comparisons, we used either a one-way ANOVA or two-way ANOVA, using GraphPad Prism (Version 10, GraphPad, San Diego, CA, USA). Statistically, significance was defined as a *p* value below 0.05.

## 3. Results

### 3.1. AIBP Deficiency Exacerbates Visual Dysfunction Induced by Oxidative Stress

**Figure 1:**
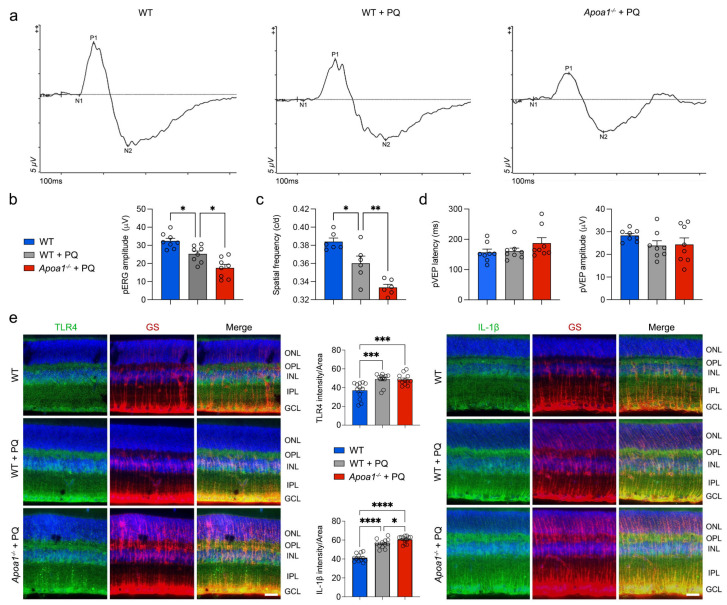
AIBP deficiency exacerbates visual dysfunction induced by oxidative stress. (**a**) Representative graphs of total recordings of pERG analysis among groups. (**b**) Quantification analysis of pERG test among groups. *N* = 8 mice. (**c**) Quantification analysis of optomotor response among groups. *N* = 8 mice. (**d**) Quantification analysis of pVEP tests among groups. *N* = 8 mice. (**e**) TLR4 and IL-1β immunohistochemistry in retina. Representative images show TLR4- and IL-1β-positive Müller glial cells in retina. Note that quantification analysis showed significant increase in IL-1β immunoreactive intensity under oxidative stress with AIBP deficiency compared with oxidative stress alone. *N* = 10 sections from middle area of retina from 3 mice. Images were taken with 20X magnification. Scale bar: 20 μm. Error bars represent SEM. Statistical significance was determined using one-way ANOVA test. * *p* < 0.05; ** *p* < 0.01; *** *p* < 0.001; **** *p* < 0.0001. pERG, pattern electroretinogram; PQ, paraquat; pVEP, pattern visual evoked potential; WT, wild-type.

AIBP expression is diminished in RGCs affected by glaucomatous damage in both human and mouse retinas [14,15]. Moreover, AIBP deficiency enhances the susceptibility of RGCs to elevated IOP, leading to impaired visual function [14,15]. Given that oxidative stress is a critical causative factor of glaucomatous retinal degeneration [3,4,5,6,7] and AIBP deficiency is linked to oxidative stress in the retina of *Apoa1bp*^−/−^ mice [15], we tested whether AIBP deficiency intensifies the impairment of visual function induced by oxidative stress. To induce oxidative stress in vivo, 10-month-old WT or *Apoa1bp*^−/−^ mice received three intraperitoneal injections of PQ within 1 week [19]. PQ is known as a ROS inducer and triggers mitochondrial superoxide production in OXPHOS complex I, resulting in oxidative stress [26]. First, we assessed RGC function through pERG analyses and observed a significant decrease in pERG amplitude in WT mice under oxidative stress (Figure 1a,b). Interestingly, our results revealed that oxidative stress further exacerbated the reduction in pERG amplitude in *Apoa1bp*^−/−^ mice compared with WT mice (Figure 1a,b). In agreement with earlier studies [15], we found that AIBP deficiency also led to a significant decrease in pERG amplitude in naïve *Apoa1bp*^−/−^ compared to WT mice (Supplementary Figure S1a,b). Next, we assessed spatial frequency using a virtual-reality optomotor system [15,19]. Consistent with pERG data, we found that AIBP deficiency intensified the reduction in spatial frequency induced by oxidative stress (Figure 1c). In addition, we assessed central vision function using a VEP analysis [15,19]. There was no statistically significant difference in pVEP latency, an indicator of disruptions or delays along visual pathways, or amplitude, an indicator of the reflection of the number of functional optic nerve fibers, among the experimental groups (Figure 1d). AIBP deficiency did not change pVEP latency or amplitude naïve *Apoa1bp*^−/−^ mice compared with WT mice (Supplementary Figure S1c). Our results suggest that, under oxidative stress conditions, AIBP deficiency exacerbates impaired visual function, likely due to the functional loss of RGCs, but causes no significant damage to the optic nerve or the conduction and processing of visual information.

Given that *Apoa1bp*^−/−^ mouse and glaucomatous human and mouse Müller glial cells showed significant increases in TLR4 and interleukin 1β (IL-1β) protein expression in human and mouse retinas [15], we tested whether AIBP deficiency enhanced oxidative stress-induced TLR4 and IL-1β protein expression in the retina. TLR4 immunoreactivity showed a remarkable increase in the WT retina under the conditions of oxidative stress alone and oxidative stress with AIBP deficiency. However, there was no statistically significant difference in TLR4 immunoreactivity between WT and *Apoa1bp*^−/−^ retinas subjected to oxidative stress alone and those with AIBP deficiency during oxidative stress (Figure 1e). On the other hand, the retina exposed to oxidative stress alone showed a notable increase in IL-1β immunoreactivity. Furthermore, AIBP deficiency showed a remarkable increase in IL-1β immunoreactivity in the retina compared with the WT retina exposed to oxidative stress (Figure 1e). In addition, both TLR4 and IL-1β immunoreactivities were increased in the processes and endfeet of glutamine synthase (GS)-positive Müller glial cells in the inner retina under the conditions of both oxidative stress in WT and oxidative stress with AIBP deficiency (Figure 1e).

### 3.2. AIBP Deficiency Intensifies Impairment of Retinal Mitochondrial Dynamics, OXPHOS Activity, and Mitochondrial Biogenesis Induced by Oxidative Stress

**Figure 2:**
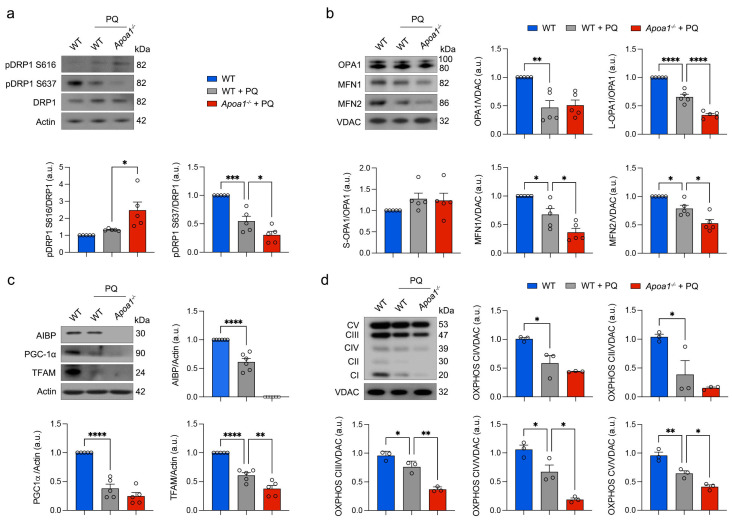
AIBP deficiency intensifies impairment of retinal mitochondrial dynamics, OXPHOS activity, and mitochondrial biogenesis induced by oxidative stress. (**a**) Total DRP1, phospho-DRP S616, and phospho-DRP1 S637 expression in retina. *N* = 3 mice. (**b**) OPA1, MFN1, and MFN2 expression in retina. *N* = 3 to 6 retinas from 3 mice. (**c**) AIBP, PGC-1α, and TFAM expression in retina. *N* = 3 mice. (**d**) OXPHOS complex expression in retina. *N* = 3 retinas from mice. Error bars represent SEM. Statistical significance was determined using one-way ANOVA test. * *p* < 0.05; ** *p* < 0.01; *** *p* < 0.001; **** *p* < 0.0001. PQ, paraquat; WT, wild-type.

AIBP deficiency compromised mitochondrial structure and function in Müller glia and RGCs in *Apoa1bp*^−/−^ mice [15]. Given that AIBP deficiency triggered dynamin-related protein 1 (DRP1) dephosphorylation at serine 637 (pDRP1 S637), an indicator of mitochondrial fragmentation, in the *Apoa1bp*^−/−^ retina [15], we investigated whether oxidative stress intensifies excessive mitochondrial fragmentation-mediated mitochondrial impairment in 10-month-old WT and *Apoa1bp*^−/−^ retinas by measuring the phosphorylation of glycogen synthase kinase 3β (GSK3β), DRP1 at serine 616 (S616), and DRP1 S637. GSK3β is a protein kinase that directly or indirectly regulates the phosphorylation of DRP1 S616 and S637 [27,28]. We observed a significant increase in pDRP1 S616 but a significant decrease in pDRP1 S637 in the *Apoa1bp*^−/−^ retina compared with the WT retina under the conditions of oxidative stress (Figure 2a; Supplementary Figure S2a). In the presence of oxidative stress, GSK3β activation was significantly enhanced in the WT retina due to GSK3β dephosphorylation (Supplementary Figure S2b). Furthermore, oxidative stress further intensified GSK3β dephosphorylation in the *Apoa1bp*^−/−^ retina (Supplementary Figure S2b). Based on our findings of the reduced protein expression of optic atrophy type 1 (OPA1) and MFN2, mitochondrial fusion proteins, in the *Apoa1bp*^−/−^ retina [15], we tested whether oxidative stress exacerbated the impairment of mitochondrial fusion activity in the *Apoa1bp*^−/−^ retina by measuring OPA1, MFN1, and MFN2 protein expression. Under the conditions of oxidative stress, OPA1, MFN1, and MFN2 protein expressions were significantly decreased in the WT retina. AIBP deficiency further exacerbated the loss of OPA1, MFN1, and 2 protein expression in the *Apoa1bp*^−/−^ retina compared with WT treated with PQ (Figure 2b). Under oxidative stress conditions, the expression of OPA1, MFN1, and MFN2 proteins was significantly reduced in the WT retina. In *Apoa1bp^−/−^* mice, this loss of OPA1, MFN1, and MFN2 expression in the retina was further aggravated compared to the PQ-treated WT retina (Figure 2b). Additionally, we observed a significant decrease in the long isoform of OPA1 (L-OPA1), which is mainly responsible for mitochondrial fusion, following a similar trend to total OPA1. In contrast, the short isoform of OPA1 (S-OPA1), associated with mitochondrial fission, remained unchanged. Based on our observation of a deteriorating mitochondrial network under the conditions of oxidative stress combined with AIBP deficiency, we further assessed whether OXPHOS function and mitochondrial biogenesis are compromised in the context of oxidative stress and AIBP deficiency. In PQ-treated WT mice, there was a significant decrease in the OXPHOS complex, as well as peroxisome proliferator-activated receptor-gamma coactivator 1α (PGC-1α) and mitochondrial transcription factor A (TFAM) protein expression in the retina, which were all further significantly reduced in PQ-treated *Apoa1bp*^−/−^ mice (Figure 2c,d).

### 3.3. Oxidative Stress Worsens Structural and Functional Impairment of Mitochondria in Müller Glial Cells Lacking AIBP

Oxidative stress induced by PQ is involved in inflammation and neuronal injury through the disruption of glial cell function [29,30]. Since glaucomatous retinal injury triggers mitochondrial dysfunction in impaired Müller glial cells linked to AIBP deficiency, oxidative stress, and inflammatory response, we tested whether oxidative stress exacerbates the structural and functional impairment of mitochondria in Müller glial cells lacking AIBP. To induce oxidative stress conditions in vitro, we employed PQ and used rMC-1 cells. The cells were treated with various concentrations of PQ (25, 50, 100, 200, 500, 1000, or 2000 μM) for 24 h (Supplementary Figure S3). We observed a PQ dose-dependent decrease in cell viability and an increase in cell death (Supplementary Figure S3a,b). Using an MTT assay to assess mitochondrial activity, we established the PQ concentration to be 500 μM which alters mitochondrial activity but does not induce cell death, as indicated by LDH release (Supplementary Figure S3b). To further assess mitochondrial dysfunction along with AIBP expression under these oxidative conditions, we also quantified MMP and measured mtROS generation at 24 h after exposure to oxidative stress. We observed that PQ-induced oxidative stress significantly induced the loss of MMP, resulting in increased mtROS generation (Supplementary Figure S3c,d). Notably, the expression level of AIBP was significantly reduced by 500 μM PQ (Supplementary Figure S3e).

**Figure 3:**
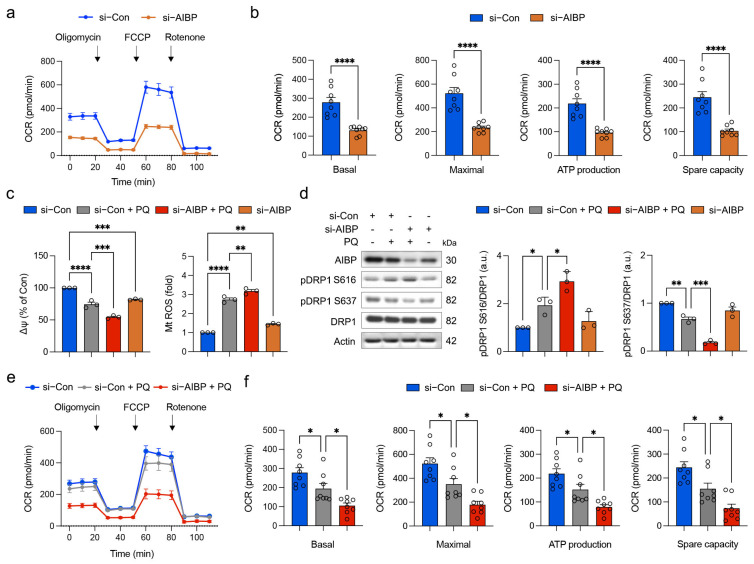
Oxidative stress worsens structural and functional impairment of mitochondria in Müller glia cells lacking AIBP. (**a**) Oligomycin A, FCCP and rotenone were sequentially added at indicated time point. Basal respiration indicates starting basal OCR and value which was set to 100%. Maximum respiration represents ratio between FCCP uncoupled OCR and basal OCR. (**b**) Quantitative analyses of basal, maximal, and ATP-linked respiration and spare respiratory capacity in rMC-1 cells. *N* = 8 replicated wells. (**c**) Quantitative analysis of MMP and mitochondrial ROS. *N* = 3 independent experiments in rMC-1 cells. (**d**) AIBP, total DRP1, phospho-DRP S616, and phospho-DRP1 S637 expression in rMC-1 cells. *N* = 3 independent experiments. (**e**) Oligomycin A, FCCP and rotenone were sequentially added at indicated time point. Basal respiration indicates starting basal OCR and value which was set to 100%. Maximum respiration represents ratio between FCCP uncoupled OCR and basal OCR. (**f**) Quantitative analyses of basal, maximal, and ATP-linked respiration and spare respiratory capacity in rMC-1 cells. *N* = 8 replicated wells. Error bars represent SEM. Statistical significance was determined using one-way ANOVA test. * *p* < 0.05; ** *p* < 0.01; *** *p* < 0.001; **** *p* < 0.0001. PQ, paraquat; FCCP, carbonyl cyanide p-trifluoromethoxyphenylhydrazone; OCR, oxygen consumption rate.

We then examined whether AIBP knockdown compromises mitochondrial respiratory function in rMC-1 cells by AIBP knockdown and Seahorse analysis. We observed a significant reduction in basal and maximal respiration, along with decreases in ATP-linked production and spare respiratory capacity in rMC-1 cells with AIBP knockdown (Figure 3a,b; Supplementary Figure S4). Subsequently, we determined the impact of oxidative stress on the impairment of MMP, a crucial factor for energy production in mitochondria, and an increase in ROS production in rMC-1 cells with AIBP deficiency. Remarkably, oxidative stress further exacerbated the reduction in MMP and the generation of ROS in rMC-1 cells with AIBP knockdown (Figure 3c; Supplementary Figure S4). Given that oxidative stress, in conjunction with AIBP knockdown, exacerbated impaired mitochondrial dynamics by increasing pDRP1 S616 and decreasing pDRP1 S637 in the retina, we then determined how oxidative stress intensifies the deregulation of mitochondrial dynamics in rMC-1 cells with AIBP knockdown. Notably, rMC-1 cells with AIBP knockdown showed a significant increase in pDRP1 S616 but a decrease in pDRP1 S637 in response to oxidative stress (Figure 3d; Supplementary Figure S4), indicating the induction of extensive mitochondrial fragmentation. Lastly, we explored the impact of oxidative stress with AIBP knockdown on mitochondrial respiratory function in rMC-1 cells. Importantly, oxidative stress triggered a significant reduction in all respiratory activities, including basal and maximal respiration, ATP-linked production, and spare respiratory capacity, in rMC-1 cells with AIBP knockdown compared with control rMC-1 cells treated with PQ (Figure 3e,f).

### 3.4. Oxidative Stress Exacerbates MAPK Activation and Apoptotic Cell Death and Inflammatory Response in Müller Glial Cells Lacking AIBP

**Figure 4:**
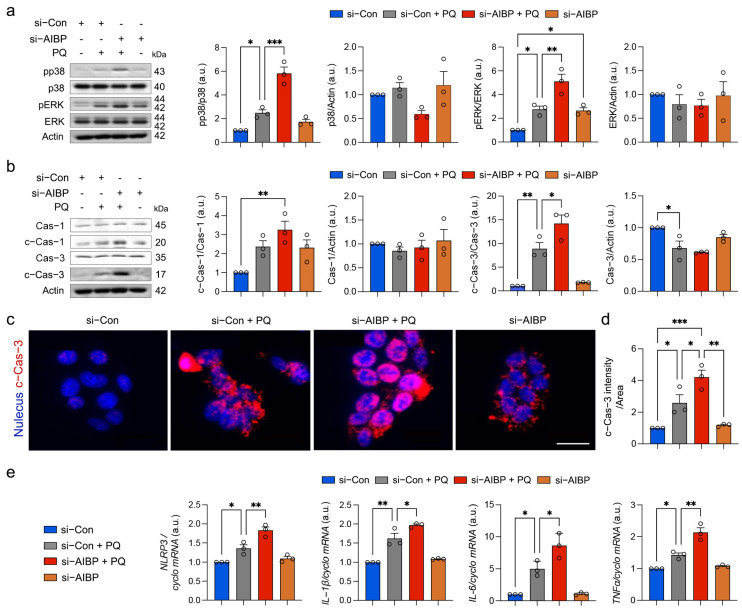
Oxidative stress exacerbates MAPK activation and apoptotic cell death and inflammatory response in Müller glia cells lacking AIBP. (**a**) p38, phospho-p38 (pp38), ERK1/2, phospho-ERK1/2 (pERK1/2) expression in rMC-1 cells. *N* = 3 independent experiments. (**b**) caspase-1, cleaved caspase-1, caspase-3, and cleaved caspase-3 expression in rMC-1 cells. *N* = 3 independent experiments. (**c**,**d**) Representative images show cleaved caspase-3-positive rMC-1 cells in the retina. Note that quantification analysis showed a significant increase in cleaved caspase-3 immunoreactive intensity in rMC-1 cells under oxidative stress with AIBP knockdown compared with control rMC-1 cells. *N* = 3 independent experiments. (**e**) Quantitative real-time PCR analysis of *Nlrp3*, *Il-1β*, *Il-6*, and *Tnfα* mRNA expression in rMC-1 cells. Error bars represent SEM. Statistical significance was determined using one-way ANOVA test. * *p* < 0.05; ** *p* < 0.01; *** *p* < 0.001. PQ, paraquat.

Given that mitogen-activated protein kinase (MAPK)-associated signaling pathways are linked to oxidative stress in Müller glial cells from glaucomatous human and mouse retinas [14,15], we tested the impact of oxidative stress on MAPK (p38 and ERK1/2) activation and apoptotic cell death in rMC-1 cells lacking AIBP. Oxidative stress significantly intensified the activation of p38 and ERK1/2 by increasing the phosphorylation of p38 (pp38) and ERK1/2 (pERK1/2) in rMC-1cells with AIBP knockdown (Figure 4a). We then examined whether oxidative stress exacerbates caspase-mediated apoptotic cell death in rMC-1 cells lacking AIBP. We observed that oxidative stress intensified the activation of caspase-1 and -3, along with increasing caspase-3 immunoreactivity, in rMC-1 cells with AIBP knockdown (Figure 4b–d), indicating an increased activation of caspase-3-mediated apoptotic cell death. The inflammasome is activated by various stimuli, including oxidative stress, leading to the activation of caspase-3 and the regulation of inflammatory responses [31]. In parallel, we found that oxidative stress exacerbated NLRP3-mediated inflammasome activation and increased the mRNA expression of the inflammatory cytokines IL-1β, IL-6, and TNF-α (Figure 4e).

### 3.5. The Administration of rAIBP Prevents Visual Dysfunction, Restores Mitochondrial Dynamics, and Enhances OXPHOS Activity in the Retina

**Figure 5:**
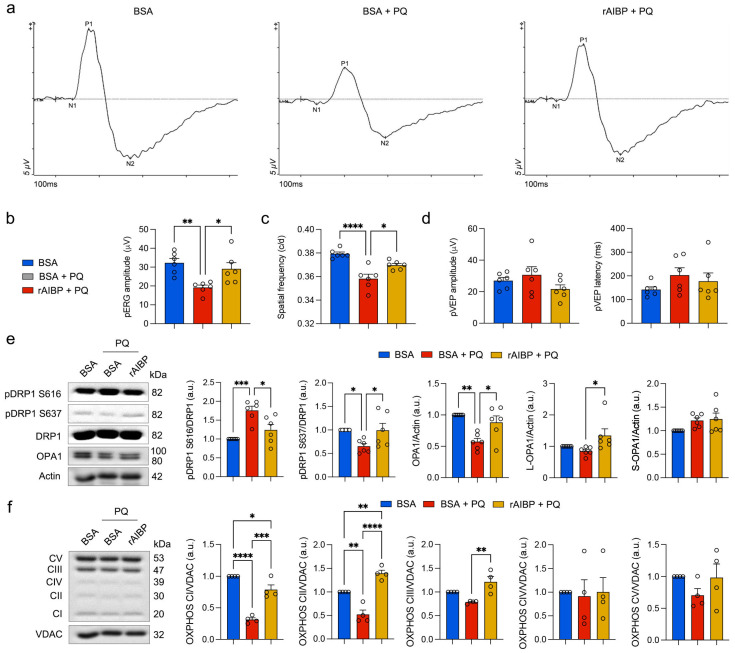
Administration of rAIBP prevents visual dysfunction, restores mitochondrial dynamics, and enhances OXPHOS activity in retina. (**a**) Representative graphs of total recordings of pERG analysis among groups. (**b**) Quantification analysis of pERG test among groups. *N* = 6 mice. (**c**) Quantification analysis of optomotor response among groups. *N* = 6 mice. (**d**) Quantification analysis of pVEP tests among groups. *N* = 6 mice. (**e**) OPA1, total DRP1, phospho-DRP S616, and phospho-DRP1 S637 expression in retina. *N* = 3 mice. (**f**) OXPHOS complex expression in retina. *N* = 3 mice. Error bars represent SEM. Statistical significance was determined using one-way ANOVA test. * *p* < 0.05; ** *p* < 0.01; *** *p* < 0.001; **** *p* < 0.0001. BSA, bovine serum albumin; PQ, paraquat; pERG, pattern electroretinogram; pVEP, pattern visual evoked potential.

Based on our observations of intensified visual dysfunction under the combined conditions of oxidative stress and AIBP deficiency (Figure 1), we hypothesized that the administration of rAIBP could potentially protect visual function in mice exposed to oxidative stress in vivo. To test the effect of rAIBP on visual function, we intravitreally injected rAIBP (1 μL, 0.5 mg/mL) or BSA (1 μL, 0.5 mg/mL) into the eyes of 10 mo old C57BL/6J mice at 2 days before the induction of oxidative stress by three IP injections for 1 week as described in the Methods Section. Similar to the experiments shown in Figure 1, we performed three different visual function tests, including pERG, pVEP, and optomotor response. Compared to oxidative stress-exposed mice treated with BSA (control), we observed that the administration of rAIBP protected RGC function as measured by pERG amplitude in mice exposed to oxidative stress (Figure 5a,b). In addition, we found a significant protection of optomotor responses through the administration of rAIBP, as evidenced by the preserved spatial frequency, in comparison to oxidative stress-exposed mice treated with BSA (Figure 5c). However, there was no statistically significant difference in pVEP amplitude and latency among the groups (Figure 5d). Because our results showed compromised mitochondrial dynamics and decreased OXPHOS activity (Figure 2), we tested whether the administration of rAIBP ameliorates the impairment of mitochondrial dynamics and OXPHOS in the retina subjected to oxidative stress. The administration of rAIBP significantly restored the expression level of the OPA1 protein and decreased pDRP1 S616 and increased pDRP1 S637 in the retina subjected to oxidative stress (Figure 5e). Moreover, we observed a substantial recovery in OXPHOS complexes I-III; however, there was no statistically significant difference in OXPHOS complexes IV and V among the experimental groups (Figure 5f).

### 3.6. Administration of rAIBP Reduced TLR4-Associated Lipid Rafts in Müller Glial Cells Exposed to Oxidative Stress

**Figure 6:**
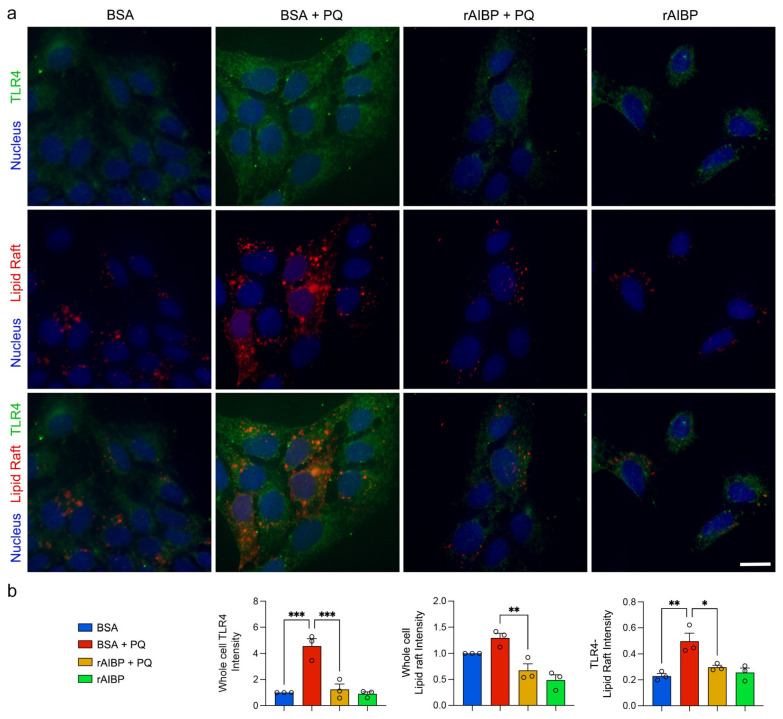
Administration of rAIBP reduced TLR4-associated lipid rafts in Müller glia exposed to oxidative stress. (**a**) Representative images of TLR4 (green)-LR (red) immunoreactivity (red). Scale bar: 10 μm. (**b**) Quantitative fluorescent intensity of TLR4-LR immunoreactivity in rMC-1 cells. a. *N* = 3 independent experiments. Scale bar: 10 μm. Error bars represent SEM. Statistical significance was determined using one-way ANOVA test. * *p* < 0.05; ** *p* < 0.01; *** *p* < 0.001. LR, lipid raft; PQ, paraquat; BSA, bovine serum albumin.

Given our observation of a substantial elevation in TLR4 expression and TLR4-associated lipid rafts in glaucomatous retinas [14], we tested whether rAIBP administration could decrease TLR4 activation and TLR4 lipid raft formation in rMC-1 cells against oxidative stress. Employing an immunofluorescence staining of TLR4-associated lipid rafts [14], we measured TLR4 expression and TLR4-associated lipid raft formation in rMC-1 cells. Under oxidative stress alone, there was a significant increase in TLR4 surface expression and TLR4-associated lipid rafts in BSA-treated rMC-1 cells (Figure 6a,b). The administration of rAIBP significantly reduced TLR4 surface expression and TLR4-associated lipid rafts in rMC-1 cells compared to BSA-treated rMC-1 cells under oxidative stress conditions (Figure 6a,b).

### 3.7. Administration of rAIBP Preserves Mitochondrial Function and Dynamics in Müller Glial Cells Exposed to Oxidative Stress

**Figure 7:**
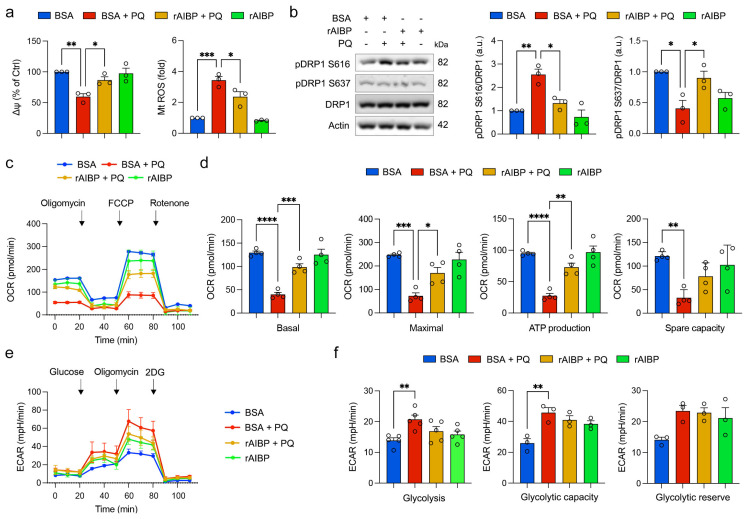
Administration of rAIBP preserves mitochondrial function and dynamics in Müller glia exposed to oxidative stress. (**a**) Quantitative analysis of MMP and mitochondrial ROS in rMC-1 cells. *N* = 3 independent experiments. (**b**) Total DRP1, phospho-DRP S616, and phospho-DRP1 S637 expression in rMC-1 cells. *N* = 3 independent experiments. (**c**) Oligomycin A, FCCP and rotenone were sequentially added at indicated time point. Basal respiration indicates starting basal OCR and value which was set to 100%. Maximum respiration represents ratio between FCCP uncoupled OCR and basal OCR. (**d**) Quantitative analyses of basal, maximal, and ATP-linked respiration and spare respiratory capacity in rMC-1 cells. *N* = 4 replicated wells. (**e**) Glucose, oligomycin A and 2DG were sequentially added at indicated time point. (**f**) Quantitative analyses of glycolysis, glycolytic capacity, and glycolytic reserve in rMC-1 cells. *N* = 5 replicated wells. Error bars represent SEM. Statistical significance was determined using one-way ANOVA test. * *p* < 0.05; ** *p* < 0.01; *** *p* < 0.001; **** *p* < 0.0001. BSA, bovine serum albumin; PQ, paraquat; FCCP, carbonyl cyanide p-trifluoromethoxyphenylhydrazone; OCR, oxygen consumption rate; ECAR, extracellular acidification rate.

To investigate the protective role of rAIBP administration in mitochondrial function and dynamics in Müller glial cells, we incubated cultured rMC-1 cells with rAIBP before inducing oxidative stress with PQ. Our findings revealed that the administration of rAIBP effectively preserved MMP and suppressed ROS generation in rMC-1 cells exposed to oxidative stress (Figure 7a). We then examined mitochondrial dynamics and found that the administration of rAIBP prevented changes in the levels of pDRP1 S616 and pDRP1 S637 in rMC-1 cells under oxidative stress conditions (Figure 7b; Supplementary Figure S5). To test mitochondrial respiratory and glycolytic function, we measured OCR and ECAR in rMC-1 cells treated with rAIBP under oxidative stress conditions. The administration of rAIBP showed a significant preservation of respiratory activities, including basal and maximal respiration, and ATP-linked production in rMC-1 cells subjected to oxidative stress (Figure 7c,d). However, there was no statistically significant difference in spare respiratory capacity in rMC-1 cells between BSA- and rAIBP-treated rMC-1 cells under oxidative stress conditions (Figure 7c,d). In addition, we found that oxidative stress triggered increases in glycolysis and glycolytic capacity (Figure 7e,f). Still, there was no statistically significant difference in glycolytic reserve in rMC-1 cells (Figure 7e,f). The administration of rAIBP did not result in a statistically significant difference in glycolysis, glycolytic capacity, or glycolytic reserve compared with BSA-treated rMC-1 cells under oxidative stress conditions (Figure 7e,f).

### 3.8. Administration of rAIBP Inhibits MAPK Activation, Apoptotic Cell Death, and Inflammatory Response in Müller Glial Cells Exposed to Oxidative Stress

**Figure 8:**
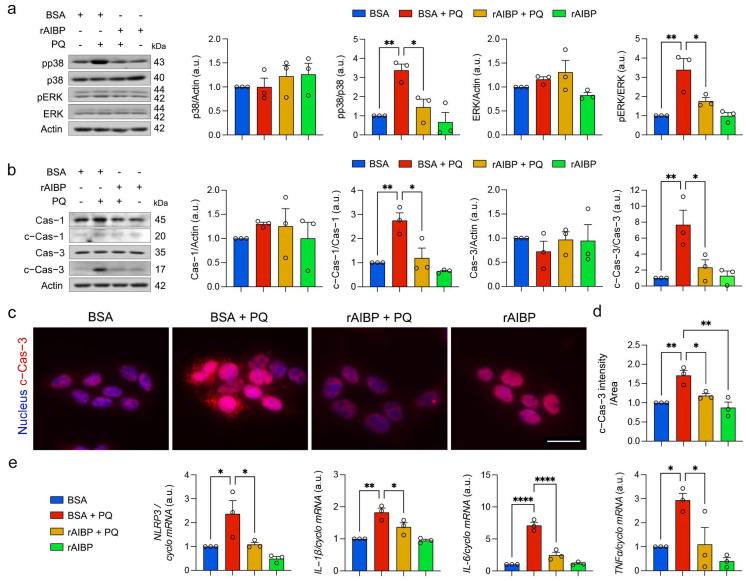
Administration of rAIBP inhibits MAPK activation, apoptotic cell death, and inflammatory response in Müller glia exposed to oxidative stress. (**a**) p38, phospho-p38 9 (pp38), ERK1/2, phospho-ERK1/2 (pERK1/2) expression in rMC-1 cells. *N* = 3 independent experiments. (**b**) caspase-1, cleaved caspase-1, caspase-3, and cleaved caspase-3 expression in rMC-1 cells. *N* = 3 independent experiments. (**c**) Representative images show cleaved caspase-3-positive rMC-1 cells. (**d**) Note that quantification analysis showed significant decrease in cleaved caspase-3 immunoreactive intensity in rMC-1 cells with rAIBP treatment compared with BSA-treated cells under oxidative stress. *N* = 3 independent experiments. (**e**) Quantitative real-time PCR analysis of *Nlrp3*, *Il-1β*, *Il-6*, and *Tnfα* mRNA expression in rMC-1 cells. Error bars represent SEM. Statistical significance was determined using one-way ANOVA test. * *p* < 0.05; ** *p* < 0.01; **** *p* < 0.0001. PQ, paraquat; BSA, bovine serum albumin.

After observing MAPK activation, caspase-1 and -3 activation, and inflammatory response in Müller glial cells lacking AIBP under oxidative stress conditions, we examined whether rAIBP administration could protect against these adverse effects in rMC-1 cells induced by oxidative stress. We found that the administration of rAIBP significantly inhibited the activation of p38 and ERK1/2, evidenced by the decreased levels of pp38 and pERK1/2 in rMC-1 cells compared with BSA-treated rMC-1 cells under oxidative stress conditions (Figure 8a). Additionally, we observed that the administration of rAIBP had a significant inhibitory effect on the activation of caspase-1 and -3, accompanied by reduced caspase-3 immunoreactivity in rMC-1 cells compared with BSA-treated rMC-1 cells under oxidative stress conditions (Figure 8b–d). This suggests an inhibition of caspase-mediated apoptotic cell death by rAIBP administration. Notably, our results also revealed that the administration of rAIBP significantly inhibited inflammasome activation and the inflammatory response, as evidenced by the decreased expression levels of the *Nlrp3*, *Il-1β*, *Il-6*, and *Tnf-α* genes in rMC-1 cells compared with BSA-treated rMC-1 cells under oxidative stress conditions (Figure 8e).

## 4. Discussion

Our recent study showed a crucial link between Müller glial cells and the increased expression of TLR4 and IL-1β in glaucomatous retinas [14,15,16]. In addition, we found that administering rAIBP protects RGCs against acute IOP elevation by suppressing cytokine production and inflammatory response [15]. Nevertheless, the precise mechanisms through which AIBP confers protection in Müller glial cells and their mitochondria have yet to be fully elucidated. This study unveils significant new findings suggesting that AIBP protects Müller glial cells against oxidative stress. In AIBP-deficient mice, the detrimental effects of oxidative stress on mitochondrial function and inflammatory reactions are exacerbated, leading to worsening vision impairment. Notably, the administration of rAIBP mitigates these consequences by reducing TLR4-associated lipid rafts. This action preserves mitochondrial dynamics and function while dampening inflammatory responses in Müller glial cells under oxidative stress, ultimately improving RGC and visual function.

Müller glial cells, the most abundant glial cells in the retina, exhibit a radial arrangement that spans the entire retinal thickness. Müller glial cells play crucial roles in the retina by establishing vital connections with retinal neurons. These functions encompass maintaining cholesterol balance, clearing waste products through phagocytosis, shielding neurons from excessive exposure to neurotransmitters like glutamate, and supplying end products from anaerobic metabolism [32,33,34,35]. Reactive glial cells, such as astrocytes and microglial cells, are closely associated with glaucomatous neuroinflammation [36]. In contrast, the involvement of reactive Müller glial cells and their mitochondrial dysfunction remains to be elucidated in glaucomatous neuroinflammation. Oxidative stress, mitochondrial dysfunction, and inflammation associated with glial activation are crucial pathogenic mechanisms in the glaucomatous retina [5,15,36,37]. Our recent findings indicated that Müller glial cells lacking AIBP display extensive mitochondrial fragmentation and reduced ATP production, resulting in Müller glial cell dysfunction linked to oxidative stress and inflammatory response [15]. Furthermore, our recent findings revealed a notable decrease in AIBP levels within glaucomatous human RGCs and Müller glial cells [14,15]. This is accompanied by increased cholesterol accumulation and TLR4-associated lipid raft formation, as well as MAPK activation, metabolic energy stress, and inflammatory responses in Müller glial cells [14,16]. Hence, these findings strongly suggested that oxidative stress could be a critical process regulated by AIBP in the context of retinal inflammation and associated with mitochondrial stress in Müller glial cells in glaucomatous neuroinflammation.

In this study, we observed a deterioration in visual function in *Apoa1bp^−/−^* mice when exposed to oxidative stress, correlating with exacerbated impairments in mitochondrial dynamics, OXPHOS activity, and mitochondrial biogenesis in the *Apoa1bp^−/−^* retina. Notably, these effects were associated with the activation of Müller glial cells, culminating in heightened inflammatory responses. Significantly, Müller glial cells lacking AIBP demonstrated increased vulnerability to degeneration, characterized by MMP loss, elevated mtROS production, excessive mitochondrial fragmentation, and compromised mitochondrial respiration, particularly under oxidative stress conditions. Oxidative stress is linked to the activation of multiple signaling pathways of MAPKs, such as p38 and ERK1/2 [38,39]. Since AIBP deficiency and glaucomatous damage activated p38 and ERK1/2 by increasing the phosphorylation of p38 and ERK1/2 in the retina [14,15], we observed that oxidative stress worsened the increase in the phosphorylation of p38 and ERK1/2 in Müller glial cells lacking AIBP. Thus, these findings strongly suggest a vicious cycle where oxidative stress initiates AIBP deficiency, leading to a cascade involving oxidative stress, AIBP deficiency, mitochondrial dysfunction, and MAPK activation in Müller glial cells. Ultimately, this cascade may trigger inflammasome activation, apoptotic cell death, and inflammatory response in oxidative stress-mediated glaucomatous neuroinflammation.

Both the restoration of AIBP expression and the administration of rAIBP significantly reduced neuroinflammation and protected RGCs in acute or chronic experimental animal models of glaucoma [14,15]. Recent evidence indicates that AIBP plays a multifaceted role, involving extracellular cholesterol efflux from the cell membrane and various intracellular functions within the mitochondria [5,11,13,15,16,40]. Specifically, intracellular AIBP has been shown to regulate autophagy/mitophagy in macrophages [13]. This study suggested that mitochondria-associated AIBP enhances mitophagy, thereby contributing to mitochondrial quality control and preventing macrophage death in atherosclerosis [13]. In this study, our findings indicated that administering rAIBP improved visual function in mice and maintained mitochondrial dynamics and function in retinal cells, including Müller glial cells, under oxidative stress conditions. These effects were supported by preventing mitochondrial fragmentation and restoring mitochondrial fusion activity and OXPHOS activity. Thus, our findings suggest that AIBP would be critical in protecting retinal mitochondria against oxidative stress. Further studies will delve into how administering rAIBP regulates mitophagy, thereby preserving mitochondrial quality control and safeguarding Müller glial cells.

Glia-driven neuroinflammation is evident in both glaucomatous human and mouse retinas, characterized by an increased expression of TLR4 and IL-1β within activated Müller glial cells [14,15]. Our observations are particularly noteworthy given that TLR4 activation typically triggers the MAPK/NFκB pathway and NLRP3 inflammasomes, resulting in an increased production of proinflammatory cytokines [41,42,43]. We found that the administration of rAIBP notably diminished TLR4-associated lipid rafts in Müller glial cells subjected to oxidative stress. Furthermore, this effect was strongly correlated with reduced MAPK activation, a suppressed NLRP3-associated inflammasome pathway, and mitigated inflammatory responses in Müller glial cells under oxidative stress conditions. Importantly, considering the established role of AIBP in reducing cholesterol deposition in glaucomatous retinas [14], our findings collectively suggest that extracellular rAIBP administration may potentially contribute to the prevention of neuroinflammation and cell death in Müller glial cells. This could be achieved through the inhibition of cholesterol deposition, reducing TLR4 inflammaraft formation, and the mitigation of mitochondrial dysfunction during oxidative stress-induced glaucomatous neuroinflammation and neurodegeneration.

Our study proposes a novel concept that oxidative stress triggers AIBP deficiency in Müller glial cells, which consequently increases the activation of TLR4 lipid rafts. This then leads to mitochondrial dysfunction, inflammasome activation, neuroinflammation, cell death, and, ultimately, vision impairment (Supplementary Figure S6). Notably, our findings support the notion that administering rAIBP counteracts these dysfunctional outcomes, safeguarding Müller glial cells. Hence, this protection may promote RGC survival and restore visual function by ameliorating glia-driven mitochondrial dysfunction and neuroinflammation in glaucoma.

## Data Availability

The original contributions presented in the study are included in the article/Supplementary Material, further inquiries can be directed to the corresponding author.

## Supplementary Materials

The following supporting information can be downloaded at: https://www.mdpi.com/article/10.3390/antiox13101252/s1, Table S1: Lists of antibodies; Table S2: Lists of PCR primers; Figure S1: AIBP deficiency exacerbates visual dysfunction induced by oxidative stress. (a) Representative graphs of total recordings of pERG analysis. (b) Quantification analysis of pERG and pVEP tests. *N* = 8 mice. Error bars represent SEM. Statistical significance determined using a two-tailed Student’s *t*-test. * *p* < 0.05. See Figure 1a,b; Figure S2: AIBP deficiency intensifies impairment of retinal mitochondrial dynamics, OXPHOS activity, and mitochondrial biogenesis induced by oxidative stress. (a) Quantification of total DRP1 expression in the retina. *N* = 3 mice. (b) Total GSK3b and phospho-GSK3b expression in the retina. *N* = 3 mice. Error bars represent SEM. Statistical significance determined using a one-way ANOVA test. * *p* < 0.05; ** *p* < 0.01. See Figure 2a; Figure S3: Oxidative stress worsens structural and functional impairment of mitochondria in Müller glial cells lacking AIBP. The rMC1 cells were treated with various concentrations of PQ (25, 50, 100, 200, 500, 1000, or 2000 μM) for 24 h. (a) Quantitative analysis of cell viability in rMC1 cells using an MTT assay. (b) Quantitative analysis of cell death in rMC1 cells using an LDH assay. (c) Quantitative analysis of MMP in rMC1 cells. (d) Quantitative analysis of mitochondrial ROS in rMC1 cells. (e) Quantitative analysis of AIBP expression in rMC1 cells. *N* = 3 independent experiments in rMC1 cells. Error bars represent SEM. Statistical significance determined using a one-way ANOVA test. * *p* < 0.05; ** *p* < 0.01; *** *p* < 0.001; **** *p* < 0.0001. See Figure 3; Figure S4: Oxidative stress worsens structural and functional impairment of mitochondria in Müller glial cells lacking AIBP. Quantification of AIBP and total DRP1 expression in the retina. *N* = 3 mice. Error bars represent SEM. Statistical significance determined using a one-way ANOVA test. * *p* < 0.05; ** *p* < 0.01. See Figure 3d; Figure S5: Administration of rAIBP preserves mitochondrial function and dynamics in Müller glial cells exposed to oxidative stress. Quantification of total DRP1 expression in the retina. *N* = 3 mice. Error bars represent SEM. See Figure 7b; Figure S6: Schematic overview of AIBP-mediated protective effect on glia-driven neuroinflammation and vision impairment. Our study proposes a novel concept that oxidative stress triggers AIBP deficiency in Müller glial cells, which consequently increases activation of TLR4-lipid raft. This then leads to mitochondrial dysfunction, inflammasome activation, neuroinflammation, cell death, and ultimately vision impairment.
