# Supplementary material for: AIBP Protects Müller Glial Cells Against Oxidative Stress-Induced Mitochondrial Dysfunction and Reduces Retinal Neuroinflammation
Source: Antioxidants (Basel). 2024 Oct 17;13(10):1252. doi: 10.3390/antiox13101252 (PMC11505583; doi:10.3390/antiox13101252)
Supplement: Supplementary file 1 [file antioxidants-13-01252-s001.zip › antioxidants-3220251-supplementary.pdf]

Supplementary Material for

AIBP protects Müller glial cells against oxidative stress-induced mitochondrial dysfunction and reduces retinal neuroinflammation

Seunghwan Choi[1], Soo-Ho Choi[2], Tonking Bastola[1], Keun-Young Kim[3], Sunsik Park[1], Robert N. Weinreb[1], Yury I. Miller[2], and Won-Kyu Ju[1,*]

[1]Hamilton Glaucoma Center, and Viterbi Family Department of Ophthalmology and Shiley Eye Institute, University of California San Diego, La Jolla, CA 92039, USA.
[2]Department of Medicine, University of California San Diego, La Jolla, CA 92039, USA.
[3]National Center for Microscopy and Imaging Research, Department of Neurosciences, University of California San Diego, La Jolla, CA 92039, USA.

***Corresponding author:** Won-Kyu Ju, Hamilton Glaucoma Center and Shiley Eye Institute, The Viterbi Family Department of Ophthalmology, University of California San Diego, 9500 Gilman Drive, La Jolla, CA 92093, USA; wju@health.ucsd.edu

This PDF file includes:

Supplementary Methods

Supplementary Figures S1 to S6

Supplementary Tables S1 and S2

Supplementary Methods

Cell viability assay

Mitochondrial activity was assessed using 3-[4, 5-dimethylthiazol-2yl]-2, 5-diphenyl tetrazolium bromide (MTT) by the manufacturer's recommendations (Cell Proliferation Kit I; Roche Diagnostics, Basel, Switzerland) as previously described. rMC1 cells were seeded into 96-well plates at a density of 1×10^4 cells/well, maintained for 1 day, and treated with the indicated concentration of PQ for 24 h. The cells were incubated with MTT solution (1 mg/ml) for 4 h at 37 °C. Then, Cell viability was determined by measuring absorbance at 550 nm using a microplate reader.

Cell death assay

Dead and membrane-ruptured cells were quantitatively measured by LDH release assay. According to the manufacturer's instructions, LDH activity in the cultured medium was assessed with Cytotoxicity Detection Kit (LDH; Sigma-Aldrich). After rMC1 cells were treated with forskolin (10 μM) and/or H_2O_2 (50 μM) for 24 h, 100 μl of the cell culture medium of each group was mixed with the 100 μl reaction mixture in a microplate followed by 30 min incubation at room temperature in dark environment. The absorbance of the samples was measured at 490 nm using a microplate leader (SpectraMAX, Molecular Devices). Two percent triton X-100-treated samples were used as positive controls. Each set of data was collected from multiple replicate dishes of each experimental group.

Supplementary Figures

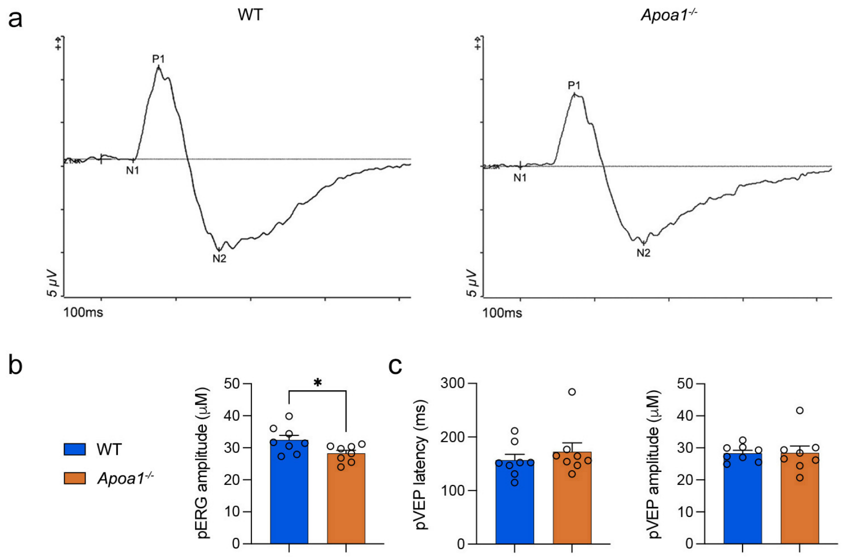

Supplemental Figure. S1. AIBP deficiency exacerbates visual dysfunction induced by oxidative stress. (**a**) Representative graphs of total recordings of pERG analysis. (**b**) Quantification analysis of pERG and pVEP tests. $N = 8$ mice. Error bars represent SEM. Statistical significance determined using a a two-tailed Student's *t*-test. $*P < 0.05$. See Fig 1a and b.

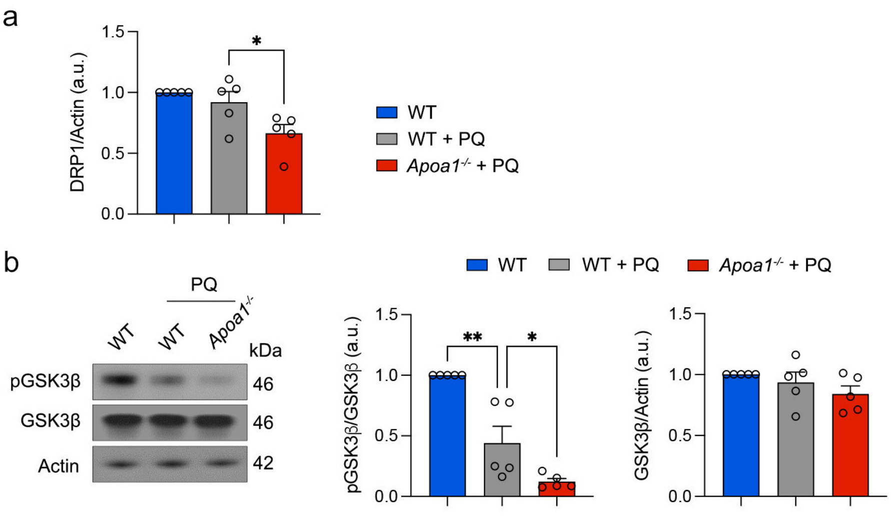

Supplemental Figure. S2. AIBP deficiency intensifies impairment of retinal mitochondrial dynamics, OXPHOS activity, and mitochondrial biogenesis induced by oxidative stress. (**a**) Quantification of total DRP1 expression in the retina. $N = 3$ mice. (**b**) Total GSK3β and phospho-GSK3β expression in the retina. $N = 3$ mice. Error bars represent SEM. Statistical significance determined using a one-way ANOVA test. *$P < 0.05$; **$P < 0.01$. See Fig. 2a.

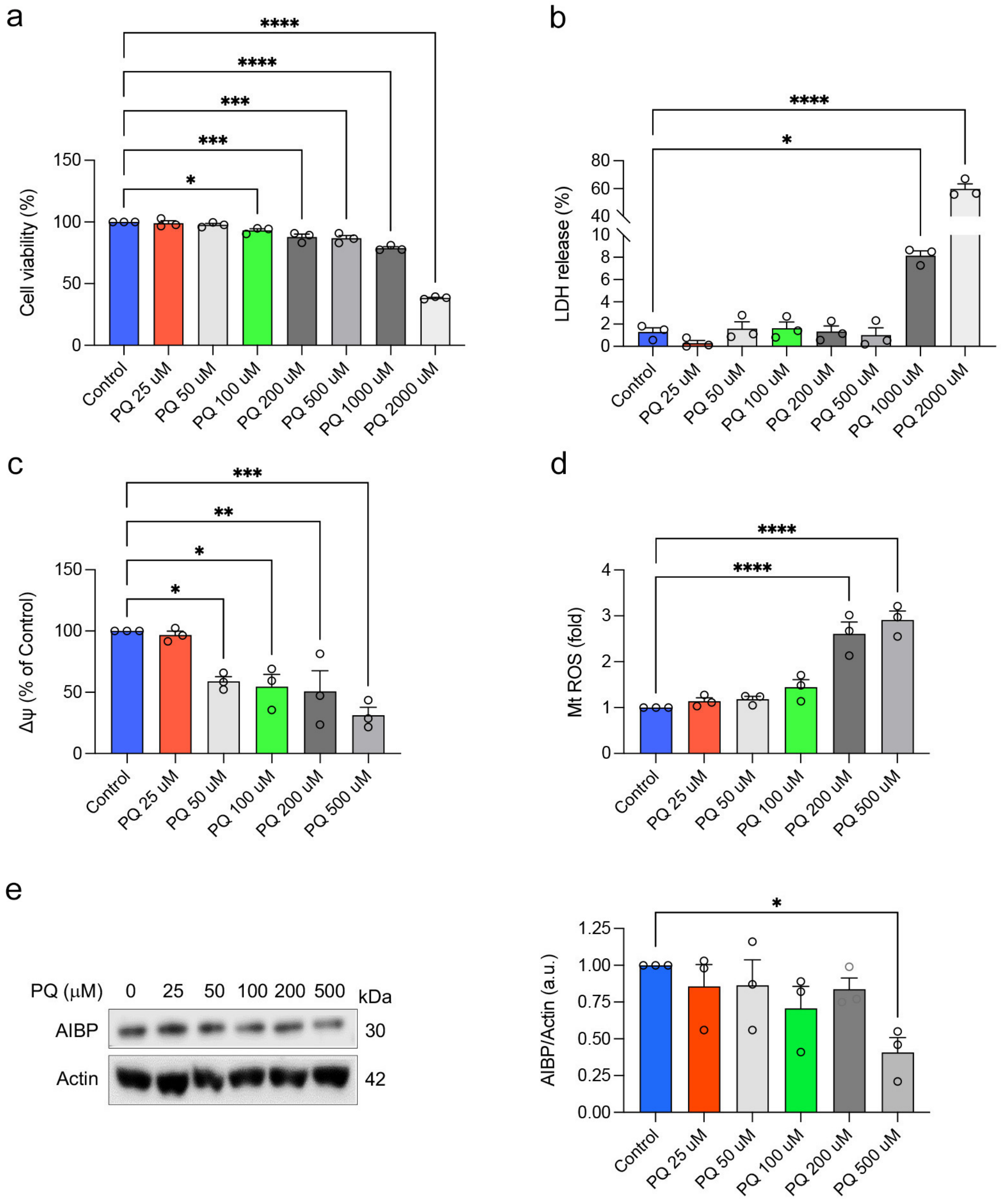

Supplementary Figure. S3. Oxidative stress worsens structural and functional impairment of mitochondria in Müller glial cells lacking AIBP. The rMC1 cells were treated with various concentrations of PQ (25, 50, 100, 200, 500, 1000, or 2000 μM) for 24 h. (**a**) Quantitative analysis of cell viability in rMC1 cells using an MTT assay. (**b**) Quantitative analysis of cell death in rMC1 cells using an LDH assay. (**c**) Quantitative analysis of MMP in rMC1 cells. (**d**) Quantitative analysis of mitochondrial ROS in rMC1 cells. (**e**) Quantitative analysis of AIBP expression in rMC1 cells. $N = 3$ independent experiments in rMC1 cells. Error bars represent SEM. Statistical significance determined using a one-way ANOVA test. *$P < 0.05$; **$P < 0.01$; ***$P < 0.001$; ****$P < 0.0001$. See Fig. 3.

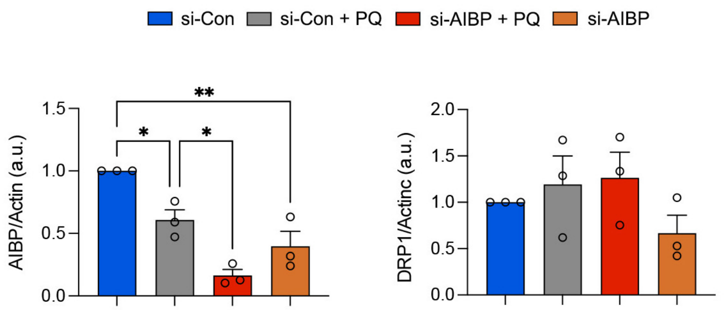

Supplemental Figure. S4. Oxidative stress worsens structural and functional impairment of mitochondria in Müller glial cells lacking AIBP. Quantification of AIBP and total DRP1 expression in the retina. *N* = 3 mice. Error bars represent SEM. Statistical significance determined using a one-way ANOVA test. $*P < 0.05$; $**P < 0.01$. See Fig. 3d.

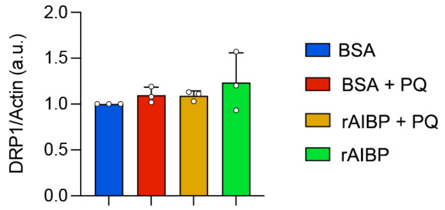

Supplementary Figure. S5. Administration of rAIBP preserves mitochondrial function and dynamics in Müller glial cells exposed to oxidative stress. Quantification of total DRP1 expression in the retina. *N* = 3 mice. Error bars represent SEM. See Fig. 7b.

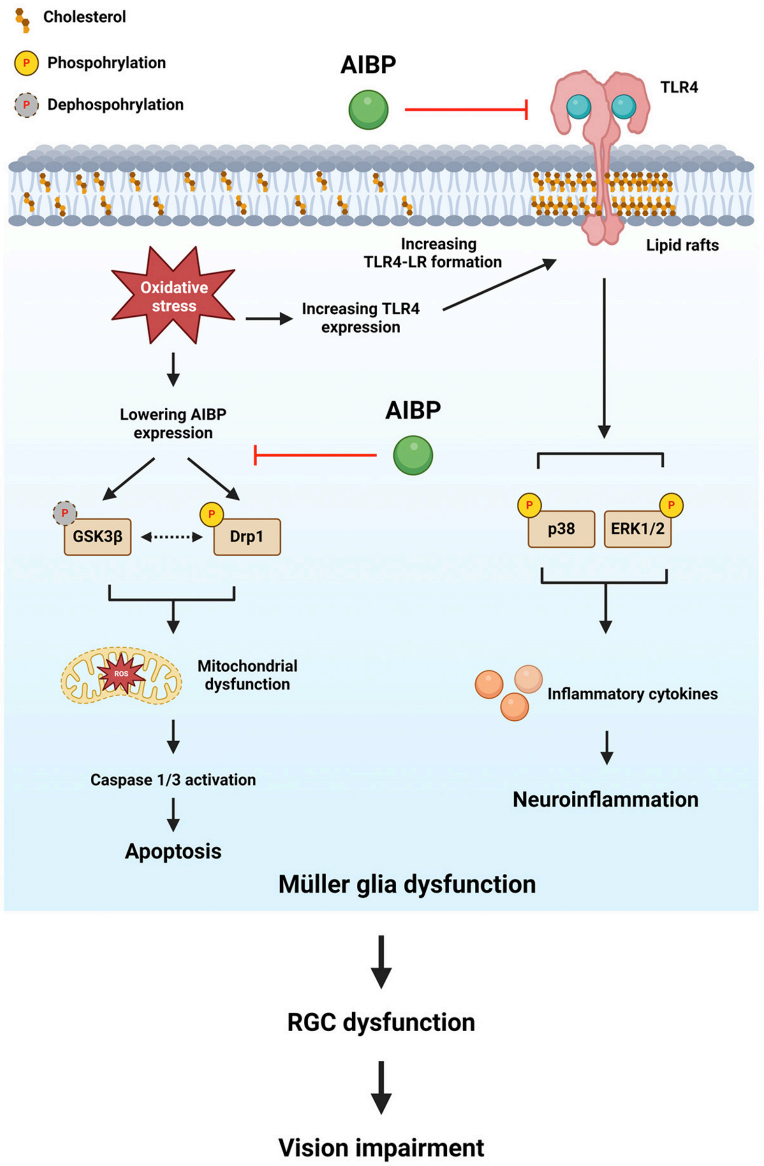

Supplementary Figure. S6. Schematic overview of AIBP-mediated protective effect on glia-driven neuroinflammation and vision impairment. Our study proposes a novel concept that oxidative stress triggers AIBP deficiency in Müller glial cells, which consequently increases activation of TLR4-lipid raft. This then leads to mitochondrial dysfunction, inflammasome activation, neuroinflammation, cell death, and ultimately vision impairment.

Supplementary Table S1. Lists of antibodies

Target antigen	Vendor or Source	Catalog No	Working concentration
AIBP (rabbit polyclonal)	Kind gift from Dr. Longhou Fang	N/A	1:5000 (WB)
Actin	Millipore	MAB1501	1:5000 (WB)
Phospho-DRP1 (Ser637)	Cell Signaling Technology	4867S	1:1000 (WB)
Phospho-DRP1 (Ser616)	Cell Signaling Technology	3455S	1:1000 (WB)
DRP1	BD science	611113	1:1000 (WB)
p38 MAPK	Cell Signaling Technology	9212S	1:1000 (WB)
Phospho-p38 MAPK (Thr180/Tyr182)	Cell Signaling Technology	9216S	1:1000 (WB)
OPA1	BD science	612607	1:1000 (WB)
Phospho-p44/42 MAPK (Erk1/2) (Thr202/Tyr204)	Cell Signaling Technology	4370S	1:1000 (WB)
p44/42 MAPK (Erk1/2)	Cell Signaling Technology	4696S	1:1000 (WB)
IL-1β	Abcam	ab9722	1:400 (WB)
Akt	Cell Signaling Technology	9272S	1:1000 (WB)
Phospho-AKT (Ser 473)	Cell Signaling Technology	4051S	1:1000 (WB)
AMPKα	Cell Signaling Technology	2532S	1:1000 (WB)
Phospho- AMPKα (Thr 172)	Cell Signaling Technology	2535S	1:1000 (WB)
GSK3beta	Cell Signaling Technology	12456S	1:1000 (WB)
Phospho-GSK3b S9	Cell Signaling Technology	5558S	1:1000 (WB)
OXPHOS	Invitrogen	458099	1:1000 (WB)
PGC-1alpha	Santa Cruz Biotechnology	sc-13067	1:1000 (WB)
TFAM	GeneTex	GTX77852	1:1000 (WB)
VDAC	Cell Signaling Technology	4866S	1:1000 (WB)
Caspase-1	Novus Biologicals	NBP1-45433	1:1000 (WB)

Caspase-3	Cell Signaling Technology	9662S	1:1000 (WB)
Cleaved Caspase-3	Cell Signaling Technology	9661S	1:1000 (WB)
MFN1	Abcam	ab57602	1:1000 (WB)
MFN2	Abcam	ab56889	1:1000 (WB)
TLR4	Proteintech	19811-1-AP	1:400 (IF)
Goat Anti-Mouse HRP	Bio-rad	1706516	1:3000 (WB)
Goat Anti-Rabbit HRP	Bio-rad	1706515	1:3000 (WB)
Alexa Fluor-488 conjugated donkey anti-rabbit IgG antibody	Invitrogen	A-21206	1:100 (IF)
CTxB-Alexa555	ThermoFisher	C34776	1:250 (IF)

Supplementary Table S2. Lists of PCR primers

Gene	Sense	Anti-sense
Rat IL-1β	TCTGACAGGCAACCACTTAC	CATCCCATACACACGGACAA
Rat NLRP3	GGAAGATGTGGACCTCAAGAAA	GATCCAAGTGATCTGCCTTCTC
Rat IL-6	AGTTGCCTTCTTGGGACTGA	ACAGTGCATCATCGCTGTTC
Rat TNF-α	ACTCGAGTGACAAGCCCGTA	GTGGGTGAGGAGCACGTAGT
Rat GAPDH	AGAACATCATCCCTGCATCC	GTCCTCAGTGTAGCCCAGGA
